# Quorum Sensing and Quorum Quenching in *Agrobacterium*: A “Go/No Go System”?

**DOI:** 10.3390/genes9040210

**Published:** 2018-04-16

**Authors:** Yves Dessaux, Denis Faure

**Affiliations:** Institute for Integrative Biology of the Cell (I2BC), CEA, CNRS, Univ. Paris-Sud, Université Paris-Saclay, Avenue de la terrasse, 91198 Gif sur Yvette CEDEX, France; denis.faure@i2bc.paris-saclay.fr

**Keywords:** *Agrobacterium*, Ti plasmid, quorum sensing, quorum quenching, lactonase, GABA, proline, (p)ppGpp

## Abstract

The pathogen *Agrobacterium* induces gall formation on a wide range of dicotyledonous plants. In this bacteria, most pathogenicity determinants are borne on the tumour inducing (Ti) plasmid. The conjugative transfer of this plasmid between agrobacteria is regulated by quorum sensing (QS). However, processes involved in the disturbance of QS also occur in this bacteria under the molecular form of a protein, TraM, inhibiting the sensing of the QS signals, and two lactonases BlcC (AttM) and AiiB that degrade the acylhomoserine lactone (AHL) QS signal. In the model *Agrobacterium* *fabrum* strain C58, several data, once integrated, strongly suggest that the QS regulation may not be reacting only to cell concentration. Rather, these QS elements in association with the quorum quenching (QQ) activities may constitute an integrated and complex “go/no go system” that finely controls the biologically costly transfer of the Ti plasmid in response to multiple environmental cues. This decision mechanism permits the bacteria to sense whether it is in a gall or not, in a living or decaying tumor, in stressed plant tissues, etc. In this scheme, the role of the lactonases selected and maintained in the course of Ti plasmid and agrobacterial evolution appears to be pivotal.

## 1. Introduction

Members of the *Agrobacterium* genus are α-proteobacteria that belong to the family Rhizobiaceae. They are plant pathogens, and may induce a disease known as crown gall on a wide range of dicotyledonous plants. The gall formation results from a genetic transformation process that relies upon the transfer of a piece of DNA, the transferred DNA (T-DNA), from the bacteria to the plant cell. In the bacteria, the T-DNA is located on the Ti (tumor-inducing) plasmid that carries most of the virulence determinants. The T-DNA transfer occurs via the activation of virulence (*vir*) genes. These genes encode a type IV secretion system (T4SS) and they are transcribed under moderately acidic conditions, mostly in response to the presence of phenolics such as acetosyringone or sinapinic acid, produced by wounded plant tissues as part of the defense reaction mechanisms (for reviews on the disease induction and genetic transformation formation process, see [1,2,3,4,5,6,7]). 

Once in the plant cell, the T-DNA is transferred to the nucleus, and it integrates into the nuclear genome. The T-DNA genes are then expressed. They encode two major functions: (i) the production of two plant hormones, i.e., auxin and cytokinins, the concomitant production of which induces the cell proliferation and the formation of the tumor [8,9,10]; and, (ii) the synthesis of low molecular weight molecules called opines, that are characteristic of *Agrobacterium*-induced overgrowths (for reviews: [4,5,6,7,8,9,10,11]). Opines play critical roles in *Agrobacterium* ecology. First, they are used by the inciting agrobacteria as specific growth substrates, the genetic determinants involved in the degradation of opines being borne on the tumour inducing (Ti) plasmid (e.g., [12,13]). Second, some opines are inducers of the conjugative transfer of the Ti plasmid which also depends on a second T4SS encoded by the Ti plasmid. Opines in tumors therefore contribute both to the multiplication of the pathogen and the dissemination of the pathogenic traits amongst the agrobacterial population, which in nature mostly consists in Ti-plasmid free cells (for reviews: [11,14,15]). 

## 2. Ti Plasmid Transfer is Regulated by Quorum Sensing and Opines

**Figure 1:**
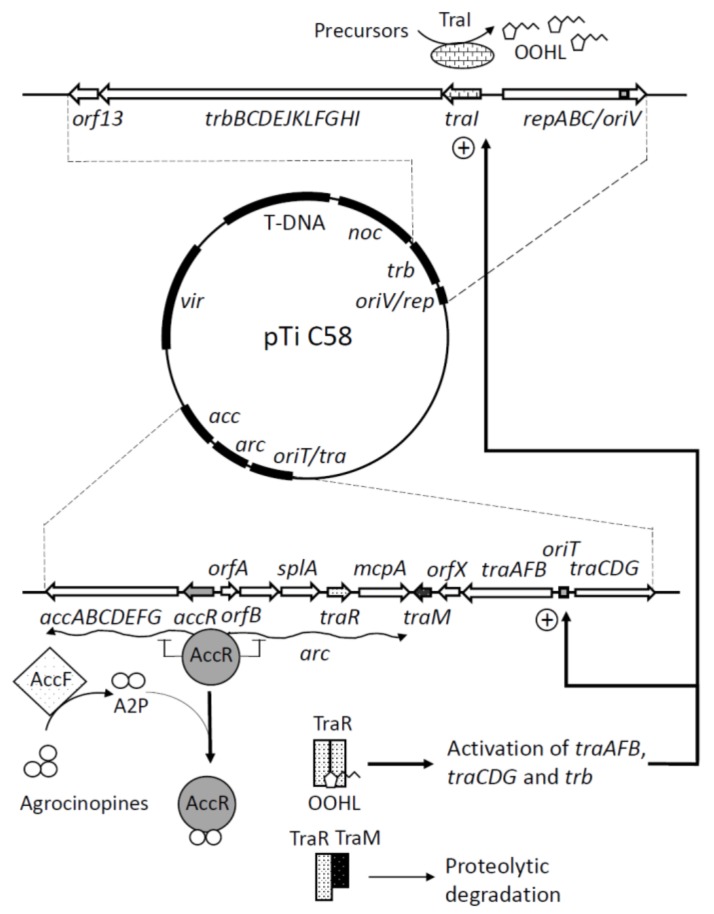
Global scheme of tumour inducing (Ti) plasmid organization and conjugation functions. The global organization of the C58 Ti plasmid is shown with magnified regions involved in Ti plasmid conjugation. In the absence of the inducing agrocinopines, the master regulator AccR prevents the transcription of both the *acc* and *arc* operons. In the presence of arabinose-2-phosphate, a catabolite of agrocinopines that binds the AccR master regulator, the repression is released, and the transcription of both the *acc* and *arc* operons occurs. Acc proteins are directing the uptake and degradation of agrocinopines. One of the genes of the *arc* operon is *traR*, a *luxR*-like gene involved in the sensing of the acylhomoserine lactone (AHL) quorum sensing (QS) signal OOHL). OOHL is synthesized by TraI, encoded by the first gene of the *trb* operon that determines the T4SS that permits the physical transfer of the Ti plasmid from one strain to another. The sensing of OOHL by TraR is antagonized by TraM that interacts with TraR to favor its proteolytic degradation. In the presence of OOHL, TraM interaction with TraR is reduced, TraR dimerizes, and becomes activated. The TraR/OOHL complex activates the transcription of the *trb* operon and that of the *traAFB* and *traCDG* operons coding the DNA transfer and replication (Dtr) system, a protein complex also known as the relaxosome. The relaxosome recognizes and cleaves the *nic* site at the origin of transfer (*oriT*) of Ti plasmids. TraG may be the coupling protein that bridges the relaxosome and its cognate T4SS. The activation of all these systems permits the conjugation of the Ti plasmid. T-DNA: transferred DNA (to plants), noc: nopaline (another opine) catabolism, oriV/rep: origine of replication and replication functions, oriT/tra: origin of conjugative transfer and conjugation function, acc: agrocinopines catabolism, vir: virulence genes, A2P: arabinose-2-phosphate, OOHL: 3-oxo-octanoylhomoserine lactone.

Ti plasmid conjugal transfer has been best described in *Agrobacterium fabrum* strain C58. In this strain, Ti plasmid transfer is also regulated by quorum sensing (QS), a cell–cell communication system responding to bacterial cell concentration via the production of the acylhomoserine lactone (AHL) signal, 3-oxo-octanoylhomoserine lactone (OOHL), that accumulates in increasing concentrations in the bacterial environment as the bacterial population grows [16,17]. In that strain, the conjugative opines are agrocinopines A and B [18]. Once bound to the catabolite of agrocinopines, arabinose-2-phosphate [19], the master repressor AccR [20], promotes the expression of two Ti plasmid-encoded operons (Figure 1). The first one, *accABCDEFG* operon, is responsible for the importation and degradation of agrocinopines [21]. A second, the *arc* operon (divergently transcribed from the former one), noticeably includes a *traR* gene that encodes TraR, a LuxR-like protein [16]. TraR exhibits two domains, one that binds DNA, the other that binds OOHL [22]. The TraR-OOHL complex activates the transcription of the *traAFB*, *traCDG*, and *trb* operons [23]. Remarkably, the first gene of the *trb* operon is *traI*, a *luxI*-like gene that encodes the OOHL signal synthase TraI [24,25]. This positive regulatory loop amplifies OOHL synthesis in the presence of agrocinopines. The *traAFB* and *traCDG* operons encode the DNA transfer and replication (Dtr) system, a protein complex also known as the relaxosome [26]. The relaxosome recognizes and cleaves the *nic* site at the origin of transfer (*oriT*) of Ti plasmids [27]. The *trb* operon determines the components of the T4SS that physically permits the conjugative transfer of Ti plasmids from one strain to another [25]. In this scheme, TraG may be the so-called coupling protein that bridges the relaxosome and its cognate T4SS [27]. The above data are related to *A. fabrum* strain C58. However, Ti plasmid conjugation and its regulation involve similar—if not identical—mechanisms and elements in other agrobacterial strains [28,29]. 

## 3. TraM Acts as a Quorum Quenching Regulator of an Unusual Quorum Sensing System

In the archetypical view of QS regulation, the above described system should respond to the cell concentration of agrobacteria. Thus, at low cell concentrations, low amounts of OOHL are produced, and the *tra* and *trb* operons, hence the *traI* genes, should not be or be only poorly expressed, even in the presence of agrocinopines. At high cell density, and in the presence of agrocinopines, the transcription of the *arc* operon is induced. TraR is therefore produced, and upon binding of OOHL, becomes activated; the *tra* and *trb* operons are expressed, thus permitting the conjugative transfer of the Ti plasmid. 

The TraR/TraI system and the encoded proteins are, however, peculiar. First, the affinity of TraR for OOHL is extremely high, in the range of 10 pM to 1 nM [30]. Second, in *Agrobacterium*, the activity of TraR is modulated by a small protein, TraM, which is also encoded by a Ti plasmid gene [31,32] (Figure 1). Indeed, TraM can bind TraR and prevent its association with OOHL [33]. Quorum quenching (QQ) refers to all processes involved in the disturbance of QS, and therefore, encompasses both the degradation of QS signals and disruption of signal sensing devices (for a review on QQ, see [34]). In this scheme, TraM may therefore be regarded as an embedded QQ regulator targeting the AHL sensor/receptor protein TraR. In support, strains defective for TraM do transfer their Ti plasmid constitutively [30,31,32]. It therefore appears that even in the absence of agrocinopines, *traR* is expressed at a level that is sufficient to bind low amounts of OOHL present in the cell and its environment [30] to activate all *tra* and *trb* operons at subquorate bacterial concentrations. Under those conditions, can the TraR/TraI system borne on the Ti plasmid be regarded as an ordinary QS system?

## 4. Two Quorum Quenching Lactonases Modulating QS and Ti Plasmid Transfer

To address the above question and fully understand the QS regulation of Ti plasmid transfer in *A. fabrum* strain C58, it is necessary to pay attention to the various QQ systems found in these bacteria. In addition to TraM QQ regulator, strain C58 exhibits two lactonases that can degrade the AHL signal OOHL. A first one is a metallo-lactonase termed AiiB [35,36]. It is encoded by the eponym gene located on the Ti plasmid. The expression of *aiiB* is not induced by short- or long-chain AHLs, nor is it by various lactones. Remarkably agrocinopines induce the expression of *aiiB* [37]. This feature remains however independent of the master regulator AccR but nevertheless requires an active agrocinopines transport system. The production of AiiB leads to a marked decreased of the OOHL that accumulates in culture supernatants of strain C58. In relation, Ti transfer frequencies measured in plant tumors are ca. 100 times higher with an *aiiB* mutant as a donor than with the wild type strain. In vitro, transconjugants of an *aiiB* defective strain appear earlier than those of the wild type strain [37]. All these data clearly demonstrate that AiiB modulates the QS-regulated transfer of the Ti plasmid. 

In strain C58, a second metallo-lactonase BlcC (formely AttM) has also been detected [35,38]. In strain C58 and several other agrobacterial isolates, the *blcC* gene is part of the *blcABC (attKLM)* operon, often located on a large megaplasmid, the At plasmid [35,39]. The *blcABC* operon is involved in the degradation of gamma-butyrolactone (GBL) to gamma-hydroxybutyrate (GHB) and semi-succinic aldehyde (SSA) [39,40]. The BlcC lactonase is able to cleave GBL and numerous AHLs, a feature related the structural similarity of these molecules. The expression of the *blcABC* operon is regulated by the repressor BlcR (AttJ) [41]; it is not affected by the presence of AHLs or opines, including agrocinopines, but it is stimulated by GBL, GHB, and SSA [40,42], though the true inducer may be SSA only [43], or SSA and GHB [41,42,43]. Plant extracts also induce the expression of BlcC [44]. 

Regarding phenolics, acetosyringone does not induce the transcription of the *blcABC* operon [45], but salicylic acid does [46]. The expression of the *blcABC* operon is also induced by gamma-aminobutyric acid (GABA) [47]. GABA is a naturally occurring non-proteinous amino acid that modulates plant growth, development, reproduction, and stress response (for reviews: [48,49,50,51]). The concentration of GABA drastically varies in plants and plant organs and tissues, especially when wounded [52]. In tomato for instance, GABA concentration ranges from 0.16 μmol/g fresh weight (FW) in tomato stem tissues to 0.57 μmol/g FW in stem tumors, and increased rapidly after wounding to reach 0.68 and 2.69 μmol/g FW in stem and tumor tissues, respectively [47]. The presence of GABA in the bacterial environment drastically increases the ability of strain C58 to inactivate OOHL. In cultures of *traR-*overexpressing mutants of strain C58 (that express *traI* in the absence of agrocinopines), OOHL reaches a concentration of 20 nM in culture supernatants, as compared with 0.5 nM in those of the wild-type, parent strain C58. In the presence of 0.5 and 1.0 mM GABA in the culture media, OOHL is not detected anymore in the supernatants of both the mutant and wild-type strain C58 [45]. In these experiments, the ability to modulate OOHL concentration appears to be clearly dependent on the presence of both a functional *blcABC* operon and the GABA transporter BraDEFG [45].

## 5. The Quorum Quenching Lactonases as Cogs to Sense Environmental Cues 

While the AHL degrading ability of BlcC has never been argued, whether there is an impact on Ti plasmid conjugation frequency has been debated. In tomato, the transfer of the Ti plasmid occurred at comparable frequency from the wild-type donor strain C58 or its *blcC* defective derivative [37]. A similar result was observed by other authors [42] who reported that transconjugants of a C58 *blcC* mutant appear ca. one week earlier than those of wild-type strain C58 in in planta conjugations, but with a similar frequency in fine. In *Arabidopsis thaliana* (Col-0 ecotype), however, the conjugation frequency was ca. 100 times higher with a *blcC* mutant of strain C58 as a donor than with the wild-type strain C58 [47].

The above discrepancy is related to the sensing of environmental cues by the bacteria, amongst which GABA and proline play critical roles. Indeed, in agrobacteria, GABA is taken up by the ABC transporter Bra and the cognate periplasmic binding protein (PBP) Atu2422 [45,53]. The PBP Atu2422 is not strictly specific for GABA. Indeed, though this uptake system does not appear to import GBL and GHB, it contributes to that of the imino/amino acids proline, alanine, and valine, as deduced from the Atu2422 structure analysis and observation that proline, as well as alanine and valine, act as competitive antagonists of GABA transport [53,54]. As a consequence, OOHL concentrations in culture supernatants of strain C58 grown in the presence of GABA are ca. 10 times lower than those measured in culture supernatant of strain C58 grown in the presence of GABA and proline, alanine or valine due to, respectively, the full or reduced activation of the *blcABC* operon [54]. 

The presented data indicate that GABA and proline ratios play important roles in the modulation of the concentration of OOHL in agrobacteria, hence, possibly on Ti plasmid conjugation. This assertion was evaluated by in planta conjugations using *A. thaliana* Col-0 plants and a mutant plant line overproducing GABA. Under those conditions, the GABA to proline ratios were 1:4 in wild type plant tumors, and 5:1 in the GABA overproducing plant tumors. Concomitantly, the Ti plasmid transfer frequencies of strain C58 Ti plasmid were ca. 100 times higher in wild-type plant tumors than in the GABA overproducing plant tumor [47]. The GABA to proline ratio may therefore be a mean for strain C58 to sense whether the bacteria is in healthy plant tissues or in a tumor. Indeed, the GABA to proline ratios in *A. thaliana* healthy and tumor tissues shifted from 3:1 in control tissues to 1:4 in tumors [47]. 

The above findings strongly suggest that the *blcC* lactonase of *Agrobacterium* may possibly play a role in the sensing of environmental clues other than the only GABA and proline concentrations. This view is strengthened by the following observations. First, the production of the BlcC lactonase activity is growth phase-dependent. The activity is only moderate throughout the log phase and becomes 8 to 10 times higher during the stationary phase. As a corollary, OOHL accumulates at the highest concentration in the late exponential growth phase [38]. Second, further investigation indicated that the BlcC lactonase activity responds to both carbon and nitrogen starvation. The underlying mechanism involves the *relA* gene that is responsible for the synthesis and degradation of the alarmones (p)ppGpp [55]. The alarmones (p)ppGpp are secondary messengers responsible for pleiotropic adaptations of bacteria (and plant chloroplasts) in response to starvation or stress, in relation with changes in RNA polymerase activity (for reviews on (p)ppGpp: [56,57]). The activation of the *blcABC* operon of *Agrobacterium* under starvation may permit the bacteria to sense whether resources are fading as this could be the case in decaying tumors (after the death of the host plant or the fall of the tumor on the soil) and consequently to prevent Ti plasmid conjugation. It may also provide the bacteria with a way to scavenge carbon from alternative sources such as GBL, GHB, and GABA, considering that this later molecule may be abundant in plant (being the major amino acid in some cases; for a review, see [51]) and can easily be converted to SSA and GHB by transamination as seen in various bacteria [58,59,60]. Interestingly, the activation of the transcription of gene *atu2422* that encodes the GABA PBP is down regulated in the stationary phase via the production of the sRNA AbcR1 [61], possibly limiting the induction of *blcABC* operon by GABA under a stress associated to nutrient deprivation. 

Last, as indicated earlier, the expression of the BlcC lactonase is stimulated by salicylic acid as much as it could be by GABA [46]. Remarkably, the concentration of the plant hormone salicylic acid drastically increases in response to mainly biotic stresses (e.g., biotrophic pathogens) but also to some abiotic ones (e.g., DNA damage) [62]. Though not demonstrated, elevated salicylic acid concentrations in stressed plants could therefore induce the expression of the BlcC lactonase, possibly leading to a modulation of the OOHL concentration and Ti conjugation activity. 

## 6. Quorum Sensing and Quorum Quenching as Two Parts of an Integrated Regulation Mechanism

The Ti plasmid conjugation is a biologically costly process. First, the Ti plasmid represents in strain C58 up to 5% of the whole genome [63,64]. Second, the induction of Ti plasmid conjugation also favors the conjugative transfer of the At plasmid [65] that may represent up to 10% of the genome [63,64]. Aside from the cost of DNA replication, the transfer involves the establishment of a T4SS that consists of a large number of proteins and requires ATP to export the DNA/protein complex [6,66]. In agreement, an *accR* mutant, which constitutively expresses the T4SS for Ti plasmid conjugation, is impaired in growth yield as compared to its wild-type parent [67]. Clearly, conjugation has to be regulated, and it is tightly regulated in *Agrobacterium*. However, control of conjugation of plasmid Ti does not only deal with metabolic cost in donors, but also to fitness advantage that is conferred by Ti plasmid (opine niche construction and exploitation) in the donors and transconjugants. 

Regulation of Ti plasmid conjugation mirrors the trade-offs between cost and gain related to Ti plasmid. As Ti plasmid confers a selective advantage to *Agrobacterium* in plant tumor, this ecological niche is optimal for vertical and horizontal propagation of Ti plasmid: the master regulator AccR and its selective interaction with opine by-product ensures opine niche-related control. Because Ti plasmid conjugation is a costly process, donors need to be in a viable state, with enough nutrients and energy resources: the lactonase BlcC, which responds to starvation [38,39,55], and stress alarmone, (p)ppGpp [55], as well as plant hormone, salicylic acid [46], both contribute to reduce Ti plasmid transfer under stressful conditions. However, the Ti plasmid donor population needs time to proliferate, hence, to be selected as the fitter population in the opine niche before transferring the Ti plasmid to recipient population: delay in OOHL synthesis and accumulation is a highly controlled process, as the QQ protein TraM, the lactonase AiiB, as well as the GABA/proline regulated lactonase BlcC, all contribute to this time control during the infection process. Both QS and QQ are regulated to optimize Ti plasmid transfer from donor *Agrobacterium*. Other authors [68] argued that OOHL degradation by BlcC was mainly accidental. In our opinion, however, under the light of the data accumulated over the past decade, the lactonase BlcC appears as one of several QQ molecular actors (TraM, BlcC, and AiiB) contributing to QS modulation in *Agrobacterium*. 

By controlling QS-signal level, QQ could also contribute to reduce QS-signal hijacking by agrobacteria (called QS-hijackers) which do not produce any QS-signal but are able to use that produced by others for activating the transfer of their own Ti plasmid. This hypothesis was recently investigated by authors [67] who reported, for the first time, the role of QQ lactonases in policing QS-signal hijacking. They showed that the QQ-lactonase may attenuate dissemination of QS-signal hijacking Ti-plasmid in co-culture assays, in which QS-signal emitting strain and QS-signal negative strain, each carrying a Ti plasmid, and a recipient strain, were mixed. The lactonase was encoded either by the QS-producing Ti-plasmid itself, by a companion plasmid in the same QS-producing donor cells, or by one in the recipient cells. In all cases, the lactonase can serve as a mechanism for controlling QS exploitation by QS signal-negative mutants [67]. 

Other arguments support our opinion that QS (TraR/TraI) and QQ (TraM/AiiB/BlcC) are not systems that only sense the quorum of a population and merely accidentally impair OOHL QS signals. First, as indicated earlier, even in the absence of agrocinopines, *traR* is expressed at a level that suffices, in the absence of TraM, to bind OOHL at subquorate bacterial concentrations [31,32,33]. Second, it is clear that BlcC and AiiB activities are modulated in response to environmental factors, such as carbon and nitrogen starvation, growth arrest, and concentration of important metabolites, such as the plant hormone salicylic acid, the stress metabolites GABA and proline, or the amino acids alanine, valine, and the agrocinopines opines [37,38,39,45,46,47,53,69,70]. 

**Figure 2:**
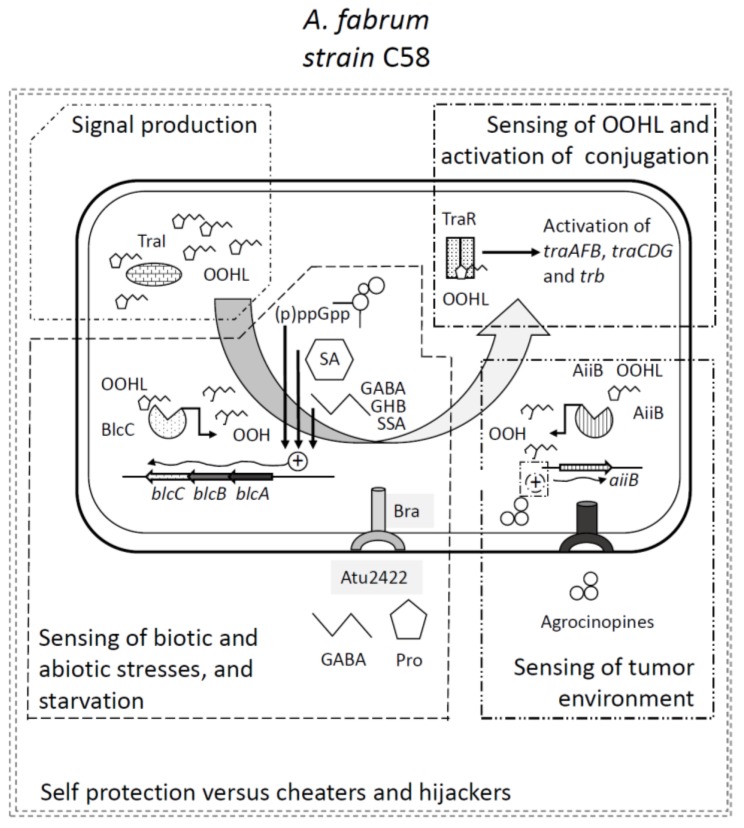
Integrated view of the quorum-sensing and quorum quenching functions in strain C58 and their involvement in the sensing of environmental parameters. The production of the QS signal OOHL results from the activity of TraI. The sensing of the signal by the sensor TraR depends on quorum quenching (QQ) activities of both the lactonases BlcC and AiiB. BlcC production is activated by various signals of environmental origins, such as the alarmones (p)ppGpp, the plant defense hormone salicylic acid, and the stress-related molecule GABA. This later enters the cell via the Atu2422 periplasmic binding protein coupled to the Bra transporter. This uptake is antagonized by molecules such as proline. The GABA to proline ratio varies drastically in healthy and crown gall tumor tissues. Salicylic acid concentration responds to mostly the presence of plant pathogens. The alarmone (p)ppGpp is involved in the regulation of the expression of multiple bacterial genes—including those of the *blcABC* operon—under starvation conditions. AiiB production is induced by the agrocinopines that also induce the conjugal transfer of the Ti plasmid. The TraI/TraR and TraM/BlcC/AiiB system defines an integrated and complex “go/no go system”, self-protected from cheaters and hijackers (see text) that finely controls the biologically costly transfer of the Ti plasmid in response to multiple environmental cues. GABA: gamma-aminobutyric acid, SA: salicylic acid.

In an effort to integrate part of these data, several regulatory models have been proposed, suggesting that the QS/QQ systems in the bacterial cell permits a fine tuning of the conjugation of the Ti plasmid throughout the *Agrobacterium* infection cycle [69,70]. *Agrobacterium* is one the bacterial models in which regulatory balance between QS and QQ was the most deeply investigated [70]. Other authors have also proposed that the QS/QQ systems of *Agrobacterium* are a way to reversibly convert bacteria, phenotypically, from plasmid recipient to donor [71]. While all these models are elegant and sound, our hypothesis (Figure 2) goes one step further and proposes that QS (TraR/TraI) and QQ (TraM/AiiB/BlcC) define an integrated regulatory “go/no go system” that finely controls the biologically costly transfer of the Ti plasmid in response to multiple environmental cues, and contributes to limit QS-hijacking. This last feature can be viewed as a self-protection of the regulatory system. This go/no go decision mechanism permits the bacteria to sense whether it is in a gall or not, in a living or decaying tumor, in stressed plant tissues, etc., and whether Ti plasmid conjugation has a chance to lead in fine to a successful event. In this scheme, the role of the lactonases is pivotal. As a support to this assertion, and in addition to the data presented above, an *blcC* mutant is less competitive than its wild-type parent in tomato and *Arabidopsis* tumors [37,47]. If indeed “*nothing in biology makes sense except in the light of evolution*” [72], the previous observation confirms that the presence of BlcC lactonase (and likely that of AiiB) has been selected and maintained in the course of Ti plasmid and agrobacterial evolution, because it is indeed beneficial to the bacteria. Experimental evolution experiments, as well as additional competition experiments performed in stressed plants or decaying tumors, could indeed help to verify the central role of lactonases in the go/no go control of Ti plasmid transfer in agrobacteria.
